# Intravascular ultrasound–guided percutaneous coronary intervention in acute coronary syndrome stratified by the TVF-ACS risk score: the IVUS-ACS trial

**DOI:** 10.1093/ehjopen/oeaf145

**Published:** 2025-10-28

**Authors:** Jing Kan, Ziwei Xi, Xiaojuan Zhang, Dandan Cai, Nailiang Tian, Xiaobo Li, Zhizhong Liu, Muhammed Anjum, Ping Xie, Xiang Chen, Hamid Sharif Khan, Xiaomei Guo, Tahir Saghir, Jing Chen, Badar Ul Ahad Gill, Ning Guo, Imad Sheiban, Fei Ye, Junjie Zhang, Feng Chen, Yongyue Wei, Gregg W Stone, Shao-Liang Chen

**Affiliations:** Nanjing First Hospital, Nanjing Medical University, Nanjing 210006, China; Center for Public Health and Epidemic Preparedness and Response, Peking University, Beijing 102627, China; Nanjing First Hospital, Nanjing Medical University, Nanjing 210006, China; Nanjing First Hospital, Nanjing Medical University, Nanjing 210006, China; Nanjing First Hospital, Nanjing Medical University, Nanjing 210006, China; Nanjing First Hospital, Nanjing Medical University, Nanjing 210006, China; Nanjing First Hospital, Nanjing Medical University, Nanjing 210006, China; Punjab Institute of Cardiology, Lahore 54000, Pakistan; Gansu Provincial People’s Hospital, Lanzhou 730000, China; Xiamen Heart Center, Xiamen University, Xiamen 361000, China; Rawalpindi Institute of Cardiology, Rawalpindi 46000, Pakistan; Tongji Hospital, Huazhong University of Science and Technology, Wuhan 430030, China; National Institute of Cardiovascular Diseases of Pakistan, Karachi 75510, Pakistan; People’s Hospital of Wuhan University, Wuhan 430060, China; Pervez Ilahi Institute of Cardiology, Lahore 54000, Pakistan; The First Affiliated Hospital of Xi’an Jiaotong University, Xi’an 710061, China; Pederzoli Hospital, Peschiera del Garda, Verona 37019, Italy; Nanjing First Hospital, Nanjing Medical University, Nanjing 210006, China; Nanjing First Hospital, Nanjing Medical University, Nanjing 210006, China; School of Public Health, Nanjing Medical University, Nanjing 211166, China; Center for Public Health and Epidemic Preparedness and Response, Peking University, Beijing 102627, China; Icahn School of Medicine at Mount Sinai, New York, NY 10029, USA; Nanjing First Hospital, Nanjing Medical University, Nanjing 210006, China

**Keywords:** Acute coronary syndrome, Percutaneous coronary intervention, Drug-eluting stent, Risk stratification, Target-vessel failure

## Abstract

**Aims:**

The IVUS-ACS trial demonstrated that intravascular ultrasound (IVUS) guidance reduces target-vessel failure (TVF) in patients with acute coronary syndromes (ACSs) undergoing percutaneous coronary intervention (PCI). Whether this benefit applies to all ACS patients across the spectrum of risk is unknown. We sought to develop a new risk score for 1-year TVF after PCI in ACS and determine whether IVUS guidance compared with angiography guidance improves outcomes in both high- and low-risk patients.

**Methods and results:**

From the angiography-guided group of the IVUS-ACS trial (*n* = 1743), the TVF-ACS risk score was developed using the least absolute shrinkage and selection operator method in a derivation group (*n* = 1288), and its robustness was assessed in an internal validation group (*n* = 455). External validation was then performed separately in the IVUS-XPL and ULTIMATE trials. Outcomes in high- and low-risk patients randomized to IVUS guidance vs. angiography guidance were then examined. Ten readily available clinical, laboratory, and angiographic variables were selected for inclusion in the TVF-ACS risk score. A cut-off value of 15.64 discriminated angiography-guided PCI patients at high-risk vs. low risk [area under the curve (AUC) 0.715, 95% confidence interval (CI) 0.653–0.777]. The AUC was similar in the validation group [0.709 (95% CI 0.630–0.788)]. High-risk patients exhibited a higher 1-year rate of TVF compared with low-risk patients [19.8 vs. 5.7%, hazard ratio (HR) 3.81, 95% CI 2.06–7.02, *P* = 0.00002]. Among 3486 randomized patients, IVUS guidance compared with angiography guidance reduced 1-year TVF in high-risk patients (6.9 vs. 17.6%; HR 0.38, 95% CI 0.24–0.59) with a lesser effect in low-risk patients (3.2 vs. 4.3%; HR 0.75, 95% CI 0.51–1.11; *P*_interaction_ = 0.02). External validation in the IVUS-XPL and ULTIMATE trials confirmed these benefits but with consistent effects in high- and low-risk patients (*P*_interactions_ = 0.49 and 0.92, respectively).

**Conclusion:**

The TVF-ACS risk score reliably stratifies ACS patients undergoing PCI into high- and low-risk groups. The benefits of IVUS guidance during PCI are most pronounced in high-risk ACS patients, although all ACS patients are likely to benefit.

## Graphical Abstract

**Graphical Abstract:**
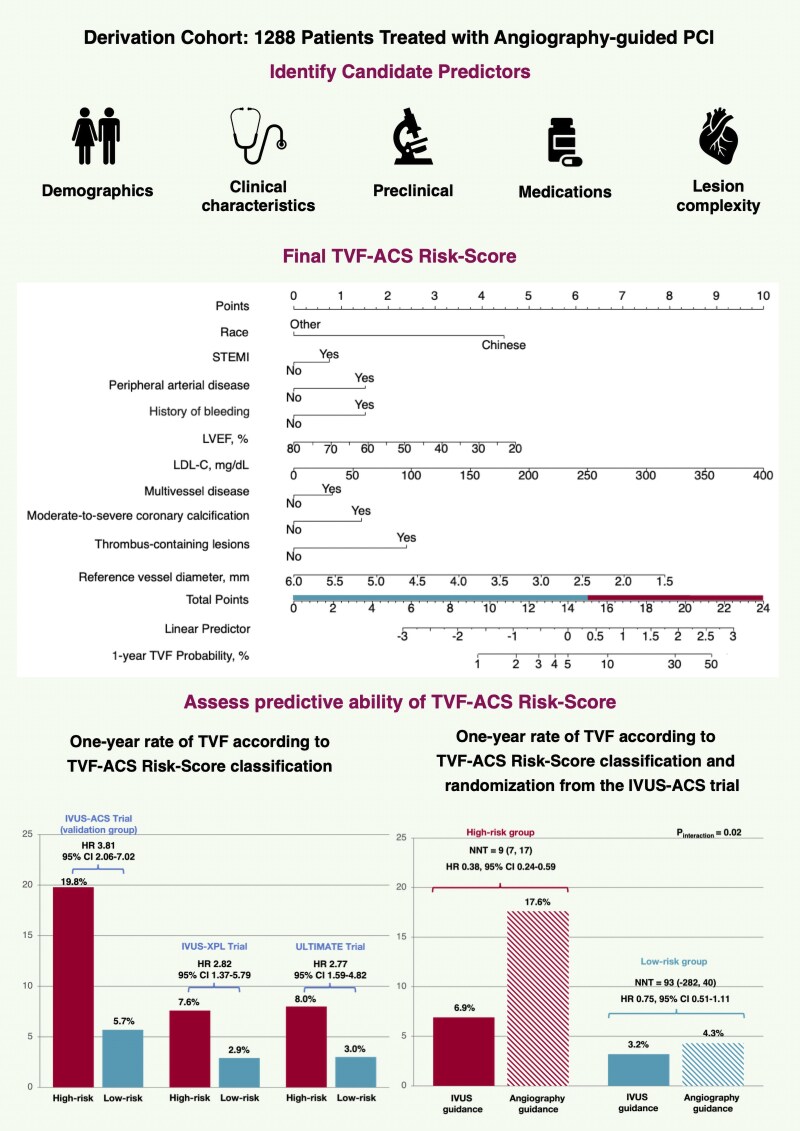

Translational perspective The rate of TVF after PCI in patients with ACS varies markedly based on clinical, laboratory and angiographic risk factors. The novel TVF-ACS risk score effectively predicts 1-year TVF risk after PCI in ACS, and identifies patients most likely to benefit from IVUS guidance during PCI. The TVF-ACS risk score may be used to discriminate patient prognosis after PCI in ACS. Moreover, while all patients are likely to benefit from IVUS guidance during PCI in ACS, the TVF-ACS risk score may be used to triage patients likely to gain the most from IVUS use in a resource-constrained environment. Further research and validation in diverse patient populations are needed to confirm the generalizability of this risk model.

## Introduction

Acute coronary syndromes (ACSs) remain the leading cause of mortality worldwide, accounting for approximately half of deaths.^1,2^ Thrombotic coronary occlusion in ACS most commonly arises from plaque rupture, endothelial erosion, and calcified nodules.^3–5^ Advancements in percutaneous coronary intervention (PCI), particularly new-generation drug-eluting stents (DESs) and contemporary anti-platelet agents, have markedly improved clinical outcomes after revascularization of culprit lesions in patients with ACS.^6–8^

Angiography provides two-dimensional imaging of the coronary artery and thus has inherent limitations in accurately assessing vessel diameter and stenosis severity, lesion length, plaque characteristics, and vessel wall involvement. Vessel overlap, lesion foreshortening, and carina shifting after bifurcation treatment are further limitations of angiography.^9^ Intravascular imaging modalities, including intravascular ultrasound (IVUS) and optical coherence tomography (OCT), have demonstrated superiority over angiography in guiding PCI, with randomized trials showing significant reductions in adverse clinical events for treatment of all-comers,^10^ long lesions,^11^ complex lesions,^12^ and bifurcation lesions.^13^

More recently, the large-scale IVUS-ACS trial demonstrated a 48% reduction in the 1-year rate of target-vessel failure (TVF) when PCI in patients presenting with an ACS were guided by IVUS rather than angiography alone.^14^ However, ACS encompasses a wide spectrum of clinical presentations, including ST-segment elevation myocardial infarction (STEMI), non-STEMI (NSTEMI), and unstable angina, as well as patients with diverse clinical risk profiles and lesion-specific characteristics.^1,8^ It is unknown whether all ACS patients uniformly benefit from IVUS guidance. Moreover, the utility of existing risk stratification tools^15–18^ that are used in routine practice to categorize ACS patients have not been demonstrated to be useful in the contemporary era of DES, potent anti-platelet agents and intravascular imaging guidance.

Accordingly, the present study using the dataset from the IVUS-ACS trial aimed to (i) develop and validate a novel instrument (the ‘TVF-ACS risk score’) to predict the 1-year risk of TVF in patients with ACS and (ii) identify the extent to which high- and low-risk patients benefit from IVUS guidance of PCI.

## Methods

### Study design and population

The IVUS-ACS (NCT02188355) was a two-stage randomized multicentre trial that evaluated the safety and effectiveness of IVUS-guided PCI compared with angiography-guided PCI in patients presenting with ACS.^14^ Patients were eligible for inclusion in the trial if they were aged 18 years or older; presented with an ACS (unstable angina, NSTEMI, or STEMI) caused by a culprit lesion in an untreated coronary artery segment within 30 days prior to randomization; and had an indication for PCI with a second-generation DES. Patients were excluded if they had a life expectancy <1 year; were intolerant of antithrombotic therapy; had severe chronic kidney disease (estimated glomerular filtration rate <20 mL/min/1.73 m^2^); or had a history of stroke within 3 months or any permanent neurological deficit or any previous intracranial bleed or intracranial disease. The IVUS-ACS trial was performed in accordance with the Declaration of Helsinki and Good Clinical Practice guidelines. The protocol was approved by the ethics committees or institutional review boards at each site, and all participants signed written informed consent.

### Outcomes and assessments

Follow-up visits were scheduled at 1 month and 1 year after discharge. Angiographic follow-up was performed only for clinical indications. Angiograms and IVUS data were analysed at independent core laboratories.

The primary endpoint was the 1-year rate of TVF, a composite of cardiac death, target-vessel myocardial infarction (TV-MI), or clinically driven target-vessel revascularization (TVR). Cardiac death was defined as any death due to the index procedure or having a proximate cardiac cause (e.g. MI, low-output failure, or fatal arrhythmia), unwitnessed death, or death of unknown cause. Myocardial infarctions were categorized as procedural vs. non-procedural; procedural MI was defined as MI occurring within 48 h of the index procedure according to the Society of Cardiac Angiography and Interventions definition,^19^ and non-procedural MI (beyond 48 h after the index procedure) was defined according to the 4th Universal Definition of Myocardial Infarction.^20^ Clinically driven revascularization included repeat PCI or coronary artery bypass graft surgery and was categorized according to its relationship to the target vessels and lesions treated during the index procedure. Secondary endpoints consisted of the individual components of the primary endpoint, TVF without procedural MI, clinically driven target lesion revascularization, major bleeding defined as Bleeding Academic Research Consortium Type 3 or 5, and stent thrombosis defined as definite or probable by Academic Research Consortium criteria.^21^ Only events confirmed by the independent clinical event adjudication committee were included in this analysis.

### Development and internal validation of the TVF-ACS risk score

**Figure 1:**
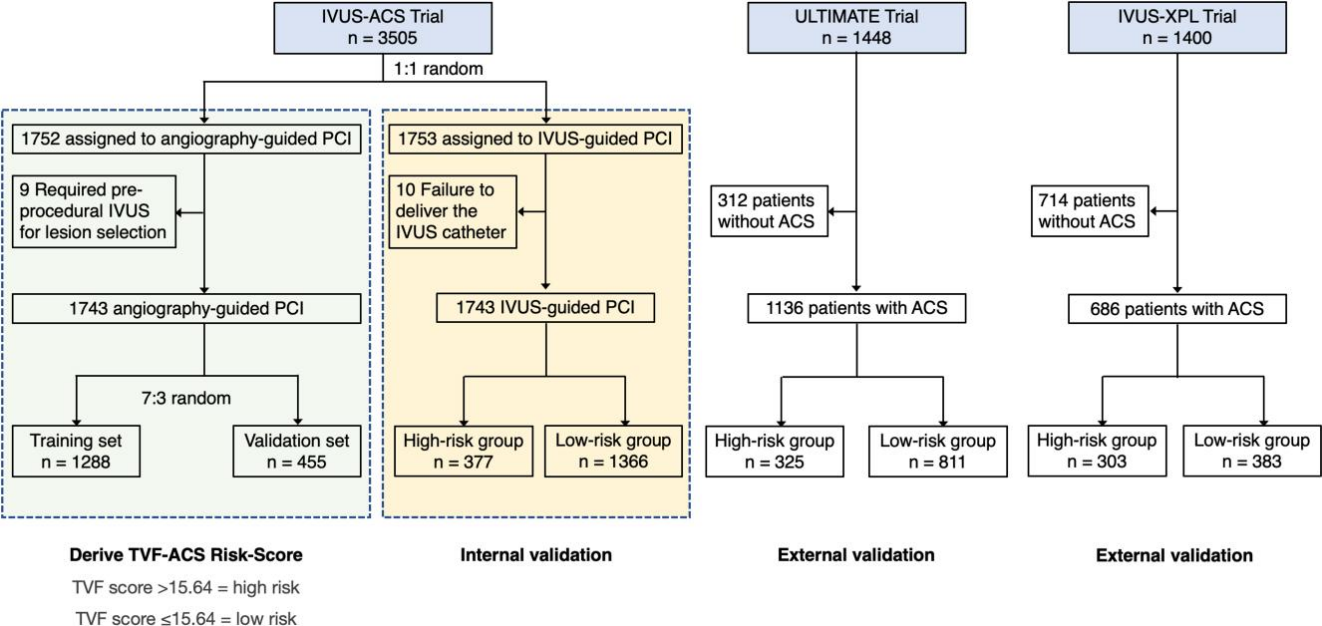
Study flowchart. Of 3505 randomized patients in the IVUS-ACS trial, 3486 patients were included in the present analysis. Among 1743 patients randomized to angiography guidance, 1288 and 455 were included in the TVF-ACS risk score derivation and validation groups respectively. The TVF-ACS risk score was then tested in 1743 patients in the intravascular ultrasound–guidance group.

The IVUS-ACS trial recruited 3505 patients between 20 August 2019 and 27 October 2022. For the present analysis, 19 patients in the IVUS-guidance group were excluded because 10 patients did not undergo IVUS assessment, and 9 patients in the angiography-guidance group were excluded in whom IVUS assessment was performed. Finally, a total of 3486 patients were included in the present study, with 1743 in each group (*Figure 1*).

The TVF-ACS risk score was developed from the angiography-guidance group. We determined the ratio to split the angiography-guided PCI group into a derivation and validation cohort using the formula √8/(√8 + 1), resulting in a 0.74:0.26 division.^19^ Thus, the 1743 participants from the angiography-guided PCI group were randomly allocated into the derivation group (*n* = 1288) and validation group (*n* = 455).

Least absolute shrinkage and selection operator (LASSO) regression with 10-fold cross-validation method was performed to identify the clinical, laboratory, functional, and angiographic indicators that exhibited differences between patients with and without TVF in the derivation group. The potential variables included are shown in *Tables 1* and *2* and Supplementary material online, *Table S1*. These initially selected variables were then further refined using stepwise Cox regression with Akaike Information Criterion assessment of goodness-of-fit. Missing values were interpolated using the K-nearest neighbours method. Using receiver operating characteristic curve analysis, the area under the curve (AUC) for the risk factor model for TVF prediction derived from Youden's index was determined, with significance testing using non-parametric bootstrap resampling (1000 iterations). The optimal threshold was determined to evaluate diagnostic specificity, sensitivity, positive and negative predictive values, and overall accuracy. Discrimination and calibration analyses were conducted to assess the performance of the model. Additionally, decision curve analyses were employed to validate the clinical utility of the nomograph. Subsequently, patients in the internal validation group were classified into low- and high-risk groups based on the TVF-ACS risk score, and the difference in 1-year TVF between the two groups was compared.

**Table 1:** Baseline clinical characteristics in patients with and without 1-year target-vessel failure in the angiography-guidance derivation cohort

	Overall (*n* = 1288)	No TVF (*n* = 1202)	TVF (*n* = 86)	*P*-value
Demographics				
Age, years	62 (54, 69)	62 (54, 69)	62 (55, 70)	0.43
Sex				0.97
Male	941 (73.1)	878 (73.0)	63 (73.3)	
Female	347 (26.9)	324 (27.0)	23 (26.7)	
Race				0.03
Chinese	1132 (87.9)	1050 (87.4)	82 (95.3)	
Non-Chinese	156 (12.1)	152 (12.6)	4 (4.7)	
Clinical characteristics				
Initial presentation				
Unstable angina	540 (41.9)	512 (42.6)	28 (32.6)	0.07
Non-STEMI	396 (30.7)	373 (31.0)	23 (26.7)	0.41
STEMI	352 (27.3)	317 (26.4)	35 (40.7)	0.004
Medical history				
Hypertension	795 (61.7)	741 (61.6)	54 (62.8)	0.83
Diabetes mellitus	402 (31.2)	372 (30.9)	30 (34.9)	0.45
Insulin treatment	109 (8.5)	96 (8.0)	13 (15.1)	0.02
Dyslipidaemia	899 (69.8)	842 (70.0)	57 (66.3)	0.46
Current smoking^a^	358 (27.8)	332 (27.6)	26 (30.2)	0.60
Chronic kidney disease	98 (7.6)	91 (7.6)	7 (8.1)	0.85
Previous PCI	144 (11.2)	132 (11.0)	12 (14.0)	0.40
Previous CABG	2 (0.2)	2 (0.2)	0 (0.0)	1.00
Previous myocardial infarction	120 (9.3)	108 (9.0)	12 (14.0)	0.13
Previous stroke	115 (8.9)	109 (9.1)	6 (7.0)	0.51
Peripheral arterial disease	60 (4.7)	52 (4.3)	8 (9.3)	0.06
Heart failure	111 (8.6)	99 (8.2)	12 (14.0)	0.07

Binary data are expressed as *n* (%). Continuous data are expressed as mean ± standard deviation or median (interquartile range).

CABG, coronary artery bypass graft surgery; PCI, percutaneous coronary intervention; STEMI, ST-segment elevation myocardial infarction.

^a^Defined as ≥100-lifetime cigarettes and still smoking at the time of enrolment; other tobacco products were not included.

**Table 2:** Angiographic and procedural characteristics in patients with and without 1-year target-vessel failure in the angiography-guidance derivation cohort

	Overall (*n* = 1288)	No TVF (*n* = 1202)	TVF (*n* = 86)	*P*-value
Number of diseased vessels	1.0 (1.0–2.0)	1.0 (1.0–2.0)	1.0 (1.0–2.0)	0.07
Single	879 (68.2)	829 (69.0)	50 (58.1)	0.04
Two	319 (24.8)	291 (24.2)	28 (32.6)	0.08
Three	90 (7.0)	82 (6.8)	8 (9.3)	0.38
Total number of lesions treated, *n*	1.0 (1.0–2.0)	1.0 (1.0–2.0)	1.0 (1.0–2.0)	0.71
Culprit lesion location				
Unprotected left main coronary artery	52 (4.0)	49 (4.1)	3 (3.5)	1.00
Left anterior descending artery	723 (56.1)	682 (56.7)	41 (47.7)	0.10
Left circumflex artery	187 (14.5)	170 (14.1)	17 (19.8)	0.15
Right coronary artery	326 (25.3)	301 (25.0)	25 (29.1)	0.41
Culprit lesion^a^ type				
True bifurcation^b^	198 (15.4)	185 (15.4)	13 (15.1)	0.95
Long or diffuse^c^	929 (72.1)	862 (71.7)	67 (77.9)	0.22
Moderate or severe calcification^d^	100 (7.8)	90 (7.5)	10 (11.6)	0.17
Thrombus containing^e^	111 (8.6)	95 (7.9)	16 (18.6)	0.001
Procedural data				
Transradial access	1241 (96.4)	1160 (96.5)	81 (94.2)	0.24
Aspiration thrombectomy used	18 (1.4)	12 (1.0)	6 (7.0)	0.001
Rotational atherectomy used	5 (0.4)	5 (0.4)	0 (0.0)	1.00
Drug-eluting stent type				
Resolute	557 (43.2)	525 (43.7)	32 (37.2)	0.24
Firehawk	656 (50.9)	613 (51.0)	43 (50.0)	0.86
Mixed	75 (5.8)	64 (5.3)	11 (12.8)	0.004
Number of stents used, *n*	1.0 (1.0–2.0)	1.0 (1.0–2.0)	1.0 (1.0–2.0)	0.21
Maximum stent diameter, mm	3.00 (3.00–3.50)	3.00 (3.00–3.50)	3.00 (2.75–3.50)	0.08
Total stent length, mm	31 (23–46)	30 (23–46)	33 (25–51)	0.12
Post-dilation performed	1199 (93.1)	1123 (93.4)	76 (88.4)	0.07
Maximum balloon pressure, atm	18 (16–18)	18 (16–18)	18 (16–20)	0.74
Contrast media, mL	150 (120–180)	150 (120–180)	155 (120–200)	0.09
Procedural time, min	30 (20–47)	30 (20–45)	39 (25–60)	0.005
Procedural success^f^	1275 (99.0)	1195 (99.4)	80 (93.0)	0.00009
Complete revascularization	1134 (88.0)	1076 (89.5)	58 (67.4)	0.0000001
Staged PCI for non-culprit lesions	92 (7.1)	85 (7.1)	7 (8.1)	0.71

Binary data are expressed as *n* (%). Continuous data are expressed as mean ± standard deviation or median (interquartile range).

IVUS, intravascular ultrasound; PCI, percutaneous coronary intervention.

^a^The lesion most likely responsible for the ACS as determined by the operator.

^b^Defined as Medina 0,1,1 or 1,1,1 bifurcation lesion with a side branch ≥2.5 mm in diameter by visual estimation.

^c^Defined as the lesion length of at least 30 mm in length by visual estimation.

^d^Defined as the angiographic presence of calcium on both sides of the vessel at the lesion site.

^e^Defined as an intraluminal filling defect seen in multiple projections.

^f^Defined as thrombolysis in myocardial infarction (TIMI) flow Grade 3, residual stenosis <20%, and absence of ≥Type B dissection, with no intra-procedural complications.

### Randomized outcomes according to the TVF-ACS risk score

After internal validation of the risk score in the angiography-guided group, patients in the IVUS-guided group from the IVUS-ACS trial were categorized as high- and low-risk based on the TVF-ACS risk score. The 1-year rates of TVF in these subgroups from all randomized patients were then compared according to randomization to IVUS guidance vs. angiography guidance of PCI, with the consistency of treatment effect examined by interaction testing.

### External validation and randomization testing of TVF-ACS risk score

The performance of TVF-ACS risk score was externally examined among patients presenting with ACS who were randomized to angiography-guided vs. IVUS-guided PCI from the IVUS-XPL and ULTIMATE trials.^10,11^ Patients were classified into high- and low-risk groups, and the risks in the 1-year rates of TVF within each group and between the randomized groups were compared.

### Statistical analysis

Categorical variables are reported as numbers and percentages and were compared using the χ^2^ test or Fisher’s exact test. Continuous variables are reported as mean ± standard deviation or median (interquartile range) if not normally distributed (as determined using the Shapiro–Wilk test and Kolmogorov–Smirnov test) and were compared using the *t*-test or the Mann–Whitney U test, respectively. Time-to-first event rates were estimated using the Kaplan–Meier method and were compared using the log-rank test. Treatment effects were estimated using Cox proportional hazards regression, with results presented as hazard ratios (HRs) and corresponding 95% CI. A 2-sided *P*-value <0.05 was considered significant for all analyses. All analyses were performed using R software (version 3.8.0).

## Results

### Baseline characteristics

Baseline clinical characteristics of the 1288 patients undergoing angiography guidance in the derivation cohort in all patients and stratified by 1-year TVF are shown in *Table 1* and Supplementary material online, *Table S1*. Target-vessel failure occurred in 86 patients (Kaplan–Meier estimated rate 6.7%). Patients with TVF were more likely to be Chinese, present with STEMI, have more insulin-treated diabetes, reduced left ventricular ejection fraction (LVEF), increased white blood cell count, liver dysfunction, and higher total and LDL-cholesterol at baseline.

Baseline angiographic and procedural characteristics of derivation cohort in all patients and stratified by 1-year TVF are shown in *Table 2* and Supplementary material online, *Table S2*. Patients with TVF were more likely to have multivessel disease, longer lesions with smaller reference vessel diameter, and more thrombus-containing lesions. The median procedural time was 9 min longer in patients with compared without TVF, and patients with TVF had lower rates of procedural success and complete revascularization.

### Derivation of the TVF-ACS risk score

Predictor selection for 1-year TVF using LASSO regression analysis in the 1288-patient angiography group derivation cohort is shown in Supplementary material online, *Figure S1*. A total of 10 variables emerged as significant predictors of 1-year TVF: race, STEMI at initial presentation, peripheral arterial disease, history of spontaneous bleeding, LDL-cholesterol, LVEF, multivessel disease, moderate or severe calcification, thrombus-containing lesions, and reference vessel diameter.

**Figure 2:**
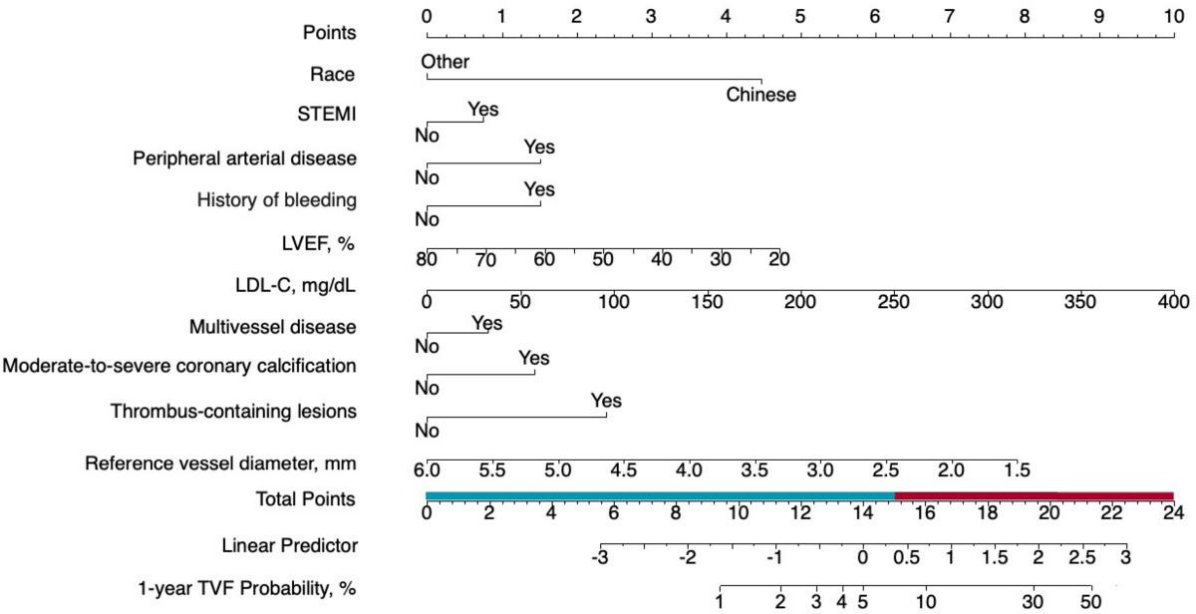
Diagnostic nomogram for prediction of 1-year target-vessel failure. Ten variables are included in the TVF-ACS risk score. Scores for each variable can be calculated from this nomogram or through an on-line calculator at: http://tvfscore.com. Patients are stratified as high risk (≥15.64 points; red bar on the total points scale) vs. low risk (<15.64 points; blue bar on the total points scale).

From these variables a nomogram was developed for TVF risk discrimination (*Figure 2*). The optimal cut-off of TVF-ACS risk score for 1-year TVF in the derivation cohort was 15.64 points. The nomogram demonstrated good discrimination with an AUC of 0.715 (95% CI 0.653–0.777; see Supplementary material online, *Figure S2A*), and calibration curves confirmed good agreement between predicted and observed TVF outcomes (Supplementary material online, *Figure S3A*). Sensitivity was 56% and specificity was 80%. Positive and negative predictive values and overall accuracy were 17, 96, and 78%, respectively.

**Figure 3:**
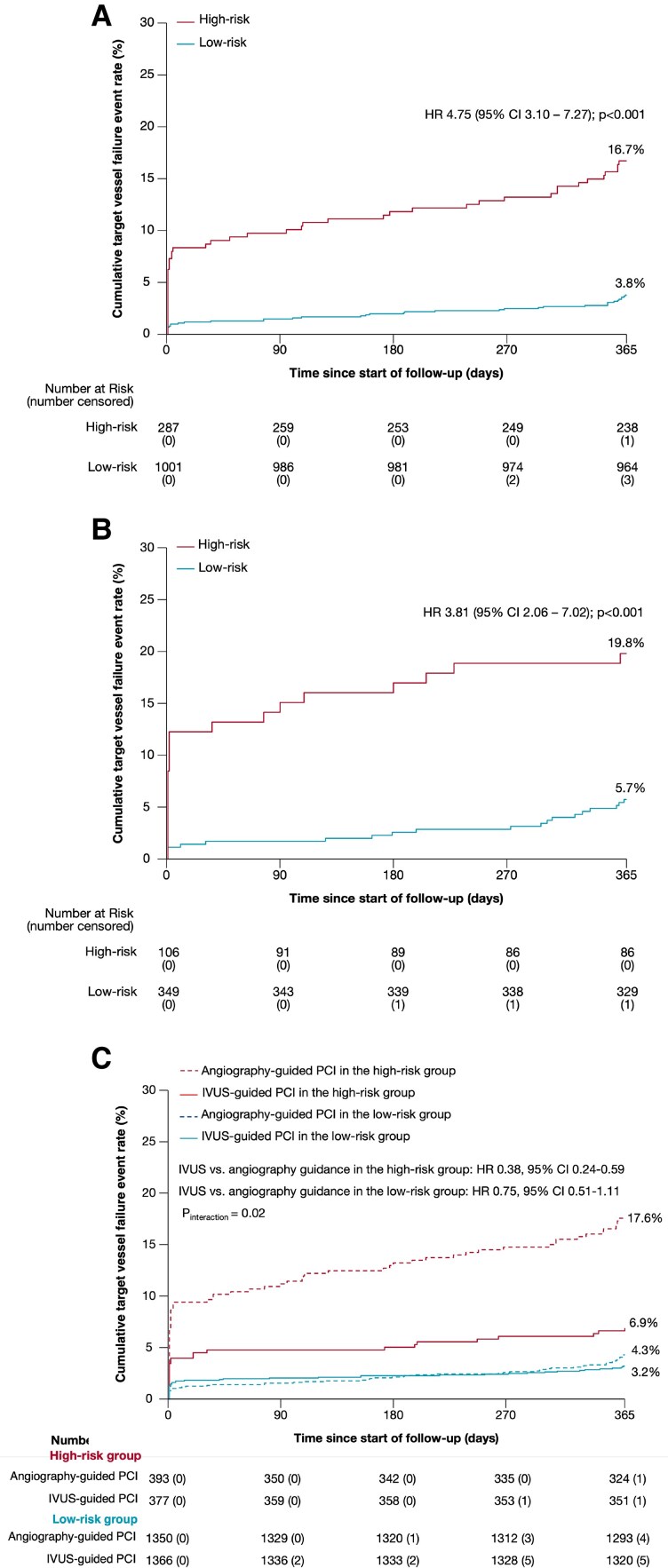
Target-vessel failure rates according to the TVF-ACS risk score from the IVUS-ACS trial. (*A*) Target-vessel failure in the angiography-guidance derivation group stratified by the TVF-ACS risk score. (*B*) Target-vessel failure in the angiography-guidance derivation group stratified by the TVF-ACS risk score. (*C*) Target-vessel failure in the high- and low-risk groups from the angiography and derivation cohorts pooled, according to randomization in each risk strata to intravascular ultrasound–guided percutaneous coronary intervention vs. angiography-guided percutaneous coronary intervention.

Among these 1288 angiography-guidance-assigned derivation group patients, using the 15.64 point cut-off, 287 (22.3%) patients were classified as high risk and 1001 (77.7%) patients were classified as low risk. High-risk compared with low-risk derivation group patients had 1-year TVF rates of 16.7 vs. 3.8%, respectively (HR 4.75, 95% CI 3.10–7.27; *P* = 0.0000000000007; *Figure 3A*).

### Internal validation of the TVF-ACS risk score

Among the 455 patients in the angiography group validation cohort, 106 (23.3%) patients were classified as high risk, and 349 (76.7%) were classified as low risk based on the 15.64 point TVF-ACS risk score cut-off. Supplementary material online, *Tables S3* and *S4* show the baseline clinical, angiographic, and procedural characteristics of low- and high-risk patients in the validation group. One-year TVF rates were 19.8% in high-risk patients compared with 5.7% in low-risk patients (HR 3.81, 95% CI 2.06–7.02; *P* = 0.00002; *Figure 3B*). Good discrimination of the TVF-ACS risk score was present in the validation cohort with an AUC of 0.709 (95% CI 0.630–0.788; see Supplementary material online, *Figure S2B*), and calibration remained high (see Supplementary material online, *Figure S3B*).

### External validation of the TVF-ACS risk score

In the IVUS-XPL trial, 686 patients with long lesions who also presented with ACS were randomized to angiography-guided PCI vs. IVUS-guided PCI. Using the 15.64 point cut-off, 303 (44.2%) patients were classified as high risk and 383 (55.8%) patients were classified as low risk by the TVF-ACS risk score. Target-vessel failure at 1 year occurred in 23 (7.6%) high-risk patients vs. 11 (2.9%) in low-risk patients (HR 2.82, 95% CI 1.37–5.79, *P* = 0.005; see Supplementary material online, *Table S5* and *Figure S4*). The AUC for the TVF-ACS risk score was 0.617 (95% CI 0.506–0.727).

In the ULTIMATE trial, 1136 ‘all-comer’ patients with few exclusion criteria who also presented with ACS were randomized to angiography-guided PCI vs. IVUS-guided PCI. Using the 15.64 point cut-off, 325 (28.6%) patients were classified as high risk and 811 (71.4%) patients were classified as low risk by the TVF-ACS risk score. Target-vessel failure at 1 year occurred in 26 (8.0%) patients in the high-risk group vs. 24 (3.0%) patients in the low-risk group (HR 2.77, 95% CI 1.59–4.82, *P* = 0.0003; see Supplementary material online, *Table S6* and *Figure S5*). The AUC for the TVF-ACS risk score was 0.621 (95% CI 0.517–0.726).

### Intravascular ultrasound guidance vs. angiography guidance in low and high TVF-ACS risk score groups

Combining the derivation and validation groups from the IVUS-ACS trial, among 1743 patients assigned to IVUS-guided PCI, 377 (21.6%) were classified as high risk and 1366 (78.4%) were classified as low risk by the TVF-ACS risk score, and among 1743 patients assigned to angiography-guided PCI, 393 (22.5%) were classified as high risk and 1350 (77.5%) were classified as low risk. Among the high-risk cohorts, 1-year TVF occurred in 26 (6.9%) patients assigned to IVUS-guided PCI vs. 69 (17.6%) patients assigned to angiography-guided PCI (HR 0.38, 95% CI 0.24–0.59). Conversely, among the low-risk cohorts, 1-year TVF occurred in 44 (3.2%) patients assigned to IVUS-guided PCI vs. 58 (4.3%) patients assigned to angiography-guided PCI (HR 0.75, 95% CI 0.51–1.11) (*P*_interaction_ = 0.02; *Figure 3C*). The number need to treat to prevent one TVF event with IVUS guidance compared with angiography guidance was 9 (95% CI 7–17) patients classified as high risk and 93 (95% CI −282 to −40) patients classified as low risk. Other 1-year outcomes stratified by risk group and randomization are shown in *Table 3*.

**Table 3:** One-year clinical outcomes in the angiography-guided and intravascular ultrasound–guided groups stratified by the TVF-ACS risk score

	Low-risk patients			High-risk patients			*P*_interaction_
	IVUS guidance (*n* = 1366)	Angiography guidance (*n* = 1350)	HR (95% CI)	IVUS guidance (*n* = 377)	Angiography guidance (*n* = 393)	HR (95% CI)	
TVF	44 (3.2)	58 (4.3)	0.75 (0.51–1.11)	26 (6.9)	69 (17.6)	0.38 (0.24–0.59)	0.02
TVF without PMI	24 (1.8)	49 (3.6)	0.48 (0.30–0.78)	14 (3.7)	40 (10.2)	0.35 (0.19–0.65)	0.43
All-cause death	8 (0.6)	11 (0.8)	0.72 (0.29–1.79)	6 (1.6)	15 (3.8)	0.41 (0.16–1.06)	0.41
Cardiac death	4 (0.3)	7 (0.5)	0.57 (0.16–1.93)	5 (1.3)	13 (3.3)	0.40 (0.14–1.11)	0.67
Target-vessel MI	28 (2.0)	22 (1.6)	1.26 (0.72–2.21)	16 (4.2)	45 (11.5)	0.36 (0.21–0.64)	0.002
Procedural	20 (1.5)	11 (0.8)	1.80 (0.86–3.75)	13 (3.4)	31 (7.9)	0.43 (0.23–0.83)	0.005
Non-procedural	8 (0.6)	11 (0.8)	0.72 (0.29–1.79)	3 (0.8)	14 (3.6)	0.22 (0.06–0.76)	0.13
TVR	16 (1.2)	35 (2.6)	0.45 (0.25–0.81)	8 (2.1)	20 (5.1)	0.41 (0.18–0.92)	0.83
TLR	14 (1.0)	27 (2.0)	0.51 (0.27–0.97)	8 (2.1)	16 (4.1)	0.51 (0.22–1.19)	0.99
Stent thrombosis	7 (0.5)	8 (0.6)	0.87 (0.31–2.38)	3 (0.8)	7 (1.8)	0.44 (0.12–1.72)	0.44

Data are number (%) of events (Kaplan–Meier estimated percentage at 1 year).

IVUS, intravascular ultrasound; PMI, procedural myocardial infarction; TLR, target-lesion revascularization; TVF, target-vessel failure; TVR, target-vessel revascularization.

Finally, while the benefits of IVUS guidance were similar, there were no significant interactions between high- vs. low-risk status and IVUS guidance vs. angiography guidance on the 1-year rates of TVF in the IVUS-XPL and ULTIMATE trials (*P*_interactions_ = 0.49 and 0.92, respectively; see Supplementary material online, *Figures S6* and *S7*).

## Discussion

In this study, we developed, internally and externally validated, and evaluated the capability of a new risk score to risk stratify outcomes in patients with ACS undergoing PCI guided by angiography and IVUS. As summarized in the *Graphical abstract*, the major findings from this report are as follows: (i) the novel TVF-ACS risk score, consisting of 10 readily available clinical, laboratory and angiographic variables, was derived and internally validated from cohorts assigned to angiography guidance in the IVUS-ACS trial, and externally validated in two independent datasets of ACS patients, the IVUS-XPL and ULTIMATE trials; (ii) the TVF-ACS risk score demonstrated good predictive power for discriminating high- vs. low-risk ACS patients; (iii) compared with angiography-guided PCI, IVUS-guided PCI reduced 1-year TVF across all risk categories, with the greatest absolute benefits observed in high-risk ACS patients.

Acute coronary syndromes encompasses a wide spectrum of patients, ranging from those with positive to negative myocardial biomarkers, without or with multiple comorbidities, and with non-complex vs. complex and extensive coronary artery disease.^1,2,6,7^ As such, the prognosis of patients with ACS treated with PCI varies greatly.^8^ However, prior risk scores have demonstrated only modest predictive accuracy in patients with ACS.^15–18,22^ Moreover, while the IVUS-ACS trial has demonstrated that by overcoming the limitations of angiography, IVUS guidance improves prognosis in ACS patients undergoing PCI,^10–12,14,23^ whether all ACS patients across risk categories derive benefit from IVUS guidance has not previously been studied.

The TVF-ACS risk score was derived and validated from patients assigned to angiography-guided PCI from the large-scale IVUS-ACS trial. A nomogram for the 1-year risk of TVF was developed from the 10 variables identified by LASSO. With a cut-off value of 15.64 points the positive and negative predictive values were 17 and 96%, and the AUC in both the derivation and internal validation datasets were similar (0.715 and 0.709, respectively). Patients in the IVUS-ACS trial with high-risk compared with low-risk scores had ∼four- to five-fold higher rates of 1-year TVF after angiography-guided PCI. Among patients with ACS in the IVUS-XPL and ULTIMATE trials, discrimination was somewhat less but still acceptable (AUC 0.617 and 0.621), and high-risk patients still had an ∼three-fold increase in 1-year TVF compared with low-risk patients.

The performance of the TVF-ACS risk score should be placed in perspective with the utility of other risk scores in patients undergoing PCI. The SYNTAX score^15^ is purely anatomy based, reflecting the extent and severity of lesion complexity, and has been most useful in stratifying outcomes between coronary artery bypass grafting and PCI.^24^ The DAPT risk score^16^ and PRECISE-DAPT scoring system^17^ predict bleeding and thrombotic risks after PCI. However, neither was designed to directly address the risk of TVF in ACS patients. The original and updated GRACE risk scores demonstrated superior discrimination over other models in predicting in-hospital and 6-month mortality and composite ischaemic events.^18,25^ As shown in Supplementary material online, *Table S7*, the TVF-ACS risk score demonstrated statistically superior discrimination and significant net reclassification improvement for the prediction of 1-year TVF compared with prior risk scores.

Among all randomized patients in the IVUS-ACS trial, IVUS guidance reduced TVF at 1 year from 7.3% with angiography guidance to 4.0%, a 45% reduction [HR 0.55 (95% CI 0.41–0.74)]. When stratified by the TVF-ACS risk score, 1-year TVF was reduced markedly with IVUS guidance compared with angiography guidance in high-risk patients (from 17.6 to 6.9%, a 62% reduction in hazard) where the effects were less dramatic in low-risk patients (from 4.3% with IVUS guidance to 3.2% with angiography guidance, a 25% reduction in hazard). The number needed to treat to prevent one event was only 9 for high-risk patients vs. 91 for low-risk patients, and a significant interaction *P*-value of 0.02 suggests a greater relative benefit of IVUS guidance in high-risk compared with low-risk patients. Conversely, in both IVUS-XPL and ULTIMATE, the relative benefits of IVUS in improving 1-year TVF were consistent in high- and low-risk patients. Whether this discordance is due to differences in sample size or patient characteristics deserves further study. Regardless, in all studies, the absolute benefit of IVUS-guided PCI compared with angiography-guided PCI was greatest in high-risk patients as identified by the TVF-ACS risk score.

### Limitations

First, the sample size for developing the TVF-ACS risk score from the study derivation group (*n* = 1288) was smaller than those from other existing risk models.^15–18,25^ Nonetheless, the performance appears superior. Whether a larger derivation group might have afforded a risk score with even greater precision is unknown. Second, the relative proportions of high-risk patients as classified by the TVF-ACS risk score varied between the IVUS-ACS, IVUS-XPL, and ULTIMATE trials (22.1, 44.2, and 28.6%, respectively), representing differences in the entry criteria for these trials. The extent to which this contributed to the somewhat lower discrimination in the IVUS-XPL and ULTIMATE trials is unknown. Moreover, we chose IVUS-XPL and ULTIMATE as the datasets to use for external validation because, like IVUS-ACS, these were randomized trials of IVUS-guided vs. angiography-guided PCI in which a large proportion of patients had ACS. External validation of the TVF-ACS risk score in other ACS trials and ‘real-world’ datasets would be of interest. Third, race (Chinese vs. other) was retained as a final variable in the TVF-ACS risk score system. Non-Chinese patients were enrolled from Pakistan, the UK, and Italy. Whether the risk score would vary in a patient population enrolled only from western sites is unknown. In this regard, the GRACE Registry primarily included white patients of European descent, and race has been strongly associated with variations in ACS risk and disparities in the quality of medical care in the USA.^26,27^ Thus, external validation of the TVF-ACS risk score (as well as the GRACE risk score 2.0) in a contemporary, multiracial ACS cohort is warranted to ensure their generalizability.^25,28^ Fourth, the present study applies only to patient outcomes after angiography-guided and IVUS-guided PCI; whether the utility of the TVF-ACS risk score would be similar with PCI guide by OCT is uncertain. Fifth, given the number of predictor variables relative to TVF events. To mitigate this, we utilized the LASSO regression method with 10-fold cross-validation, a technique specifically designed to enhance model stability in such scenarios. The consistent performance of the model upon internal and external validation further suggests that overfitting was unlikely to be a major factor. Finally, the types of IVUS devices and the expertise of operators were not standardized across the participating centres, which may introduce a degree of procedural heterogeneity.

## Conclusions

The novel TVF-ACS risk score effectively stratifies ACS patients undergoing PCI into high- and low-risk groups. Compared with angiography-guided PCI, IVUS-guided PCI in patients with ACS reduces the 1-year rate of TVF across the spectrum of risk, with the most pronounced benefits observed in high-risk patients.

## Supplementary Material

oeaf145_Supplementary_Data

## Data Availability

The patient-level data supporting this study are not publicly available to protect patient privacy. Collaborative use of the data may be permitted upon reasonable request directed to the corresponding author, Shao-Liang Chen.
