# Supplementary material for: Intravascular ultrasound–guided percutaneous coronary intervention in acute coronary syndrome stratified by the TVF-ACS risk score: the IVUS-ACS trial
Source: Eur Heart J Open. 2025 Oct 28;5(6):oeaf145. doi: 10.1093/ehjopen/oeaf145 (PMC12625814; doi:10.1093/ehjopen/oeaf145)
Supplement: oeaf145_Supplementary_Data [file oeaf145_supplementary_data.docx]

**Supplemental Appendix**

**IVUS-guided PCI in ACS Stratified by a Novel Risk-Score: The IVUS-ACS trial**

|  |  | **Page** |
| --- | --- | --- |
| Table S1 | Additional baseline characteristics and discharge medications in patients with and without 1-year TVF in the angiography guidance derivation cohort | 2 |
| Table S2 | Quantitative coronary angiographic analysis in patients with and without 1-year TVF in the angiography guidance derivation cohort | 4 |
| Table S3 | Baseline clinical characteristics in the angiography guidance validation group | 5 |
| Table S4 | Angiographic and procedural characteristics in the angiography guidance validation group | 7 |
| Table S5 | Performance of the TVF-ACS Risk-Score among ACS patients in the IVUS-XPL trial | 9 |
| Table S6 | Performance of the TVF-ACS Risk-Score among ACS patients in the ULTIMATE trial | 10 |
| Table S7 | Model discrimination and calibration performance of various risk scores for one-year target vessel failure compared with the internal validation dataset from IVUS-ACS trial | 11 |
| Figure S1 | Predictor selection using LASSO regression analysis with tenfold cross-validation | 12 |
| Figure S2 | Discrimination of the TVF-ACS Risk-Score for 1-year TVF | 13 |
| Figure S3 | Calibration of the TVF-ACS Risk-Score for 1-year TVF | 14 |
| Figure S4 | Performance of the TVF-ACS Risk-Score in the IVUS-XPL trial | 15 |
| Figure S5 | Performance of the TVF-ACS Risk-Score in the ULTIMATE trial | 16 |
| Figure S6 | One-year TVF in high-risk and low-risk patient groups randomized to angiography guidance vs. IVUS guidance in the IVUS-XPL trial | 17 |
| Figure S7 | One-year TVF in high-risk and low-risk patient groups randomized to angiography guidance vs. IVUS guidance in the ULTIMATE trial | 18 |

**Table S1. Additional baseline characteristics and discharge medications in patients with and without 1-year TVF in the angiography guidance derivation cohort**

|  | **Overall**  **(n=1288)** | **No TVF**  **(n=1202)** | **TVF**  **(n=86)** | **p value** |
| --- | --- | --- | --- | --- |
| Height, cm | 168 (161, 172) | 168 (161, 172) | 167 (161, 170) | 0.28 |
| Weight, kg | 70 (63, 78) | 70 (63, 78) | 68 (62, 75) | 0.14 |
| Body mass index, kg/m^2^ | 25.1 (23.1, 27.4) | 25.2 (23.2, 27.4) | 24.7 (22.9, 27.0) | 0.43 |
| Systolic blood pressure, mmHg | 130 (120, 142) | 130 (120, 142) | 130 (120, 149) | 0.89 |
| Diastolic blood pressure, mmHg | 78 (70, 85) | 78 (70, 85) | 76 (69, 85) | 0.31 |
| Heart rate, bpm | 72 (68, 80) | 72 (68, 80) | 72 (64, 82) | 0.75 |
| Family history of CAD | 120 (9.3) | 113 (9.4) | 7 (8.1) | 0.70 |
| Previous spontaneous bleeding | 17 (1.3) | 15 (1.2) | 2 (2.3) | 0.32 |
| COPD | 19 (1.5) | 17 (1.4) | 2 (2.3) | 0.37 |
| Anemia | 46 (3.6) | 41 (3.4) | 5 (5.8) | 0.23 |
| Liver disease | 65 (5.0) | 57 (4.7) | 8 (9.3） | 0.07 |
| Malignancy | 20 (1.6） | 20 (1.7) | 0 (0.0) | 0.64 |
| Left ventricular ejection fraction, % | 62 (56, 65) | 62 (56, 65) | 60 (49, 65) | 0.04 |
| **Laboratory findings** |  |  |  |  |
| White blood cells, x 10^9^/L | 6.92 (5.77, 8.54) | 6.88 (5.74, 8.47) | 7.65 (6.40, 10.26) | <0.001 |
| Red blood cells, x 10^12^/L | 4.55 (4.17, 4.90) | 4.55 (4.19, 4.90) | 4.51 (4.05, 5.00) | 0.53 |
| Hemoglobin, g/L | 138 (127, 148) | 138 (127, 148) | 137 (122, 152) | 0.92 |
| Platelet, x 10^9^/L |  |  |  |  |
| SGPT, IU | 22 (16, 34) | 22 (15, 33) | 23 (17, 41) | 0.12 |
| SGOT, IU | 23 (18, 34) | 23 (18, 34) | 25 (19, 73) | 0.04 |
| Serum creatinine, mg/dL | 0.80 (0.69, 0.93) | 0.80 (0.69, 0.93) | 0.81 (0.66, 0.93) | 0.90 |
| eGFR, ml/min/m^2^ | 92 (78, 109) | 92 (78, 109) | 89 (79, 106) | 0.59 |
| Fasting glucose, mg/dL | 100.9 (89.3, 122.0) | 101.0 (89.5, 122.1) | 100.4 (87.5, 120.2) | 0.82 |
| Total cholesterol, mg/dL | 152.4 (128.0, 182.9) | 151.6 (127.2, 182.1) | 170.9 (136.5, 202.2) | 0.01 |
| LDL-cholesterol, mg/dL | 88.9 (68.1, 115.6) | 87.8 (67.7, 114.1) | 105.6 (76.2, 127.2) | 0.003 |
| **Medications at discharge after percutaneous coronary intervention** | | | | |
| Aspirin | 1288 (100.0) | 1202 (100.0) | 86 (100.0) | 1.00 |
| Ticagrelor | 1288 (100.0) | 1202 (100.0) | 86 (100.0) | 1.00 |
| β blocker | 619 (48.1) | 585 (48.7) | 34 (39.5) | 0.10 |
| ACEI or ARB | 596 (46.3) | 559 (46.5) | 37 (43.0) | 0.53 |
| Calcium channel antagonist | 341 (26.5) | 321 (26.7) | 20 (23.3) | 0.48 |
| Statin | 1085 (84.2) | 1029 (85.6) | 56 (65.1) | <0.001 |
| Proton pump inhibitor | 727 (56.4) | 690 (57.4) | 37 (43.0) | 0.009 |

Binary data are expressed as n (%). Continuous data are expressed as mean ± standard deviation or median (interquartile range). TVF, target vessel failure; ACEI denotes angiotensin converting enzyme inhibitor; ARB, angiotensin receptor blocker; CAD, coronary heart disease; COPD, chronic obstructive pulmonary disease; eGFR, estimated glomerular filtration rate; LDL, low density lipoprotein; SGPT, serum glutamic-pyruvic transaminase; SGOT, serum glutamic-oxaloacetic transaminase.

**Table S2. Quantitative coronary angiographic analysis in patients with and without 1-year TVF in the angiography guidance derivation cohort**

|  | **Overall**  **(n=1288)** | **No TVF**  **(n=1202)** | **TVF**  **(n=86)** | **p value** |
| --- | --- | --- | --- | --- |
| **Baseline quantitative coronary angiography** | | | | |
| Reference vessel diameter, mm | 3.00 (2.60, 3.40) | 3.00 (2.61, 3.41) | 2.73 (2.31, 3.16) | 0.008 |
| Minimal lumen diameter, mm | 1.08 (0.66, 1.41) | 1.09 (0.67, 1.43) | 0.78 (0.00, 1.17) | <0.001 |
| Diameter stenosis, % | 59.9 (52.5, 69.0) | 59.7 (52.3, 69.0) | 61.3 (54.0, 69.0) | 0.75 |
| Lesion length, mm | 26.5 (18.4, 37.2) | 26.4 (18.3, 36.4) | 35.1 (21.7, 46.4) | <0.001 |
| **Post-procedure quantitative coronary angiography** | | | | |
| Reference vessel diameter, mm | 3.15 (2.77, 3.56) | 3.15 (2.77, 3.57) | 3.04 (2.71, 3.46) | 0.15 |
| Minimal lumen diameter, mm | 2.71 (2.34, 3.06) | 2.71 (2.35, 3.07) | 2.69 (2.29, 2.91) | 0.09 |
| Acute gain, mm | 1.68 (1.23, 2.20) | 1.66 (1.23, 2.19) | 1.90 (1.41, 2.53) | 0.03 |
| Diameter stenosis, % | 14.0 (7.8, 20.3) | 14.1 (7.8, 20.3) | 13.2 (9.5, 20.6) | 0.29 |

Binary data are expressed as n (%). Continuous data are expressed as mean ± standard deviation or median (interquartile range). TVF, target vessel failure.

**Table S3. Baseline clinical characteristics in the angiography guidance validation group**

|  | **High-risk**  **(n=106)** | **Low-risk**  **(n=349)** | **P value** |
| --- | --- | --- | --- |
| Age, years | 65 (54, 71) | 64 (54, 69) | 0.26 |
| Sex |  |  | 0.15 |
| Male | 87 (82.1) | 263 (75.4) |  |
| Female | 19 (17.9) | 86 (24.6) |  |
| Race |  |  | 0.001 |
| Chinese | 104(98.1) | 301 (86.2) |  |
| Non-Chinese | 2 (1.9) | 48 (13.8) |  |
| Height, cm | 170 (165, 174) | 168 (160, 171) | 0.008 |
| Weight, kg | 72 (65, 80) | 70 (62, 77) | 0.02 |
| Body mass index, kg/m^2^ | 25.4 (23.2, 27.7) | 24.8 (22.9, 27.1) | 0.30 |
| Systolic blood pressure, mmHg | 129 (112, 144) | 130 (124, 140) | 0.04 |
| Diastolic blood pressure, mmHg | 78 (70, 88) | 78 (70, 84) | 0.61 |
| Heart rate, bpm | 72 (68, 84) | 72 (67, 80) | 0.24 |
| **Clinical characteristics** |  |  |  |
| Initial presentation |  |  |  |
| Unstable angina | 17 (16.0) | 168 (48.1) | <0.001 |
| Non-STEMI | 27 (25.5) | 111 (31.8) | 0.21 |
| STEMI | 62 (58.5) | 70 (20.1) | <0.001 |
| Medical history |  |  |  |
| Hypertension | 63 (59.4) | 224 (64.2) | 0.38 |
| Diabetes mellitus | 39 (36.8) | 108 (30.9) | 0.26 |
| Insulin treatment | 10 (9.4) | 25 (7.2) | 0.44 |
| Dyslipidemia | 74 (69.8) | 241 (69.1) | 0.88 |
| Current smoking* | 40 (37.7) | 87 (24.9) | 0.010 |
| Chronic kidney disease | 9 (8.5) | 18 (5.2) | 0.20 |
| Previous PCI | 9 (8.5) | 23 (6.6) | 0.50 |
| Previous CABG | 0 (0.0) | 2 (0.6) | 1.00 |
| Previous myocardial infarction | 8 (7.5) | 25 (7.2) | 0.89 |
| Previous stroke | 17 (16.0) | 36 (10.3) | 0.11 |
| Peripheral arterial disease | 9 (8.5) | 12 (3.4) | 0.06 |
| Heart failure | 12 (11.3) | 14 (4.0) | 0.005 |
| Family history of coronary heart disease | 6 (5.7) | 32 (9.2) | 0.25 |
| Previous spontaneous bleeding | 6 (5.7) | 5 (1.4) | 0.02 |
| Chronic obstructive pulmonary disease | 1 (0.9) | 5 (1.4) | 1.00 |
| Anemia | 3 (2.8) | 15 (4.3) | 0.78 |
| Liver disease | 9 (8.5) | 7 (2.0) | 0.004 |
| Malignancy | 2 (1.9) | 5 (1.4) | 0.67 |
| Left ventricular ejection fraction, % | 57 (48, 64) | 64 (60, 66) | <0.001 |
| **Laboratory findings** |  |  |  |
| White blood cells, x 10^9^/L | 7.5 (6.3, 9.7) | 6.6 (5.7, 8.3) | 0.001 |
| Red blood cells, x 10^12^/L | 4.4 (4.2, 4.9) | 4.5 (4.2, 4.9) | 0.74 |
| Hemoglobin, g/L | 137 (128, 149) | 139 (127, 147) | 0.86 |
| Platelet, x 10^9^/L | 207 (171, 257) | 206 (169, 252) | 0.78 |
| SGPT, IU | 27 (18, 48) | 23 (15, 33) | 0.02 |
| SGOT, IU | 27 (19, 97) | 21 (17, 33) | <0.001 |
| Serum creatinine, mg/dL | 0.79 (0.70, 0.92) | 0.81 (0.70, 0.93) | 0.50 |
| eGFR, ml/min/m^2^ | 94 (80, 108) | 90 (78, 106) | 0.24 |
| Fasting glucose, mg/dL | 110.0 (97.0, 148.9) | 100.1 (88.4, 122.6) | <0.001 |
| Total cholesterol, mg/dL | 181.4 (150.6, 206.5) | 146.2 (120.7, 170.9) | <0.001 |
| LDL-cholesterol, mg/dL | 115.0 (89.7, 139.2) | 81.6 (64.2, 103.6) | <0.001 |
| **Medications at discharge after percutaneous coronary intervention** | | | |
| Aspirin | 106 (100.0) | 349 (100.0) | 1.00 |
| Ticagrelor | 106 (100.0) | 349 (100.0) | 1.00 |
| β blocker | 47 (44.3) | 171 (49.0) | 0.40 |
| ACEI or ARB | 50 (47.2) | 162 (46.4) | 0.89 |
| Calcium channel antagonist | 24 (22.6) | 89 (25.5) | 0.55 |
| Statin | 81 (76.4) | 303 (86.8) | 0.01 |
| Proton pump inhibitor | 57 (53.8) | 184 (52.7) | 0.85 |

Binary data are expressed as n (%). Continuous data are expressed as mean ± standard deviation or median (interquartile range).

ACEI denotes angiotensin converting enzyme inhibitor; ARB, angiotensin receptor blocker; CABG, coronary artery bypass graft surgery; eGFR, estimated glomerular filtration rate, LDL, low density lipoprotein, PCI, percutaneous coronary intervention; SGPT, serum glutamic-pyruvic transaminase; SGOT, serum glutamic-oxaloacetic transaminase; STEMI, ST-segment elevation myocardial infarction. *Defined as ≥100 lifetime cigarettes and still smoking at the time of enrolment; other tobacco products were not included.

**Table S4. Angiographic and procedural characteristics in the angiography guidance validation group**

|  | **High-risk**  **(n=106)** | **Low-risk**  **(n=349)** | **P value** |
| --- | --- | --- | --- |
| Number of diseased vessels | 1.0 (1.0, 2.0) | 1.0 (1.0, 2.0) | 0.001 |
| Single | 62 (58.5) | 260 (74.5) | 0.002 |
| Two | 30 (28.3) | 73 (20.9) | 0.11 |
| Three | 14 (13.2) | 16 (4.6) | 0.002 |
| Total number of lesions treated, n | 1.0 (1.0, 2.0) | 1.0 (1.0, 1.0) | 0.02 |
| Culprit lesion location |  |  |  |
| Unprotected left main coronary artery | 4 (3.8) | 12 (3.4) | 0.77 |
| Left anterior descending artery | 59 (55.7) | 188 (53.9) | 0.75 |
| Left circumflex artery | 13 (12.3) | 60 (17.2) | 0.23 |
| Right coronary artery | 30 (28.3) | 89 (25.5) | 0.57 |
| Culprit lesion* type |  |  |  |
| True bifurcation† | 18 (17.0) | 46 (13.2) | 0.32 |
| Long or diffuse§ | 71 (67.0) | 240 (68.8) | 0.73 |
| Moderate or severe calcification¶ | 15 (14.2) | 14 (4.0) | <0.001 |
| Thrombus-containing‖ | 36 (34.0) | 15 (4.3) | <0.001 |
| Procedural data |  |  |  |
| Transradial access | 101 (95.3) | 338 (96.8) | 0.55 |
| Aspiration thrombectomy used | 8 (7.5) | 1 (0.3) | <0.001 |
| Rotational atherectomy used | 1 (0.9) | 0 (0.0) | 0.23 |
| Drug-eluting stent type |  |  |  |
| Resolute | 30 (28.3) | 150 (43.0) | 0.007 |
| Firehawk | 73 (68.9) | 182 (52.1) | 0.002 |
| Mixed | 3 (2.8) | 17 (4.9) | 0.59 |
| Number of stents used, n | 1.0 (1.0, 2.0) | 1.0 (1.0, 2.0) | 0.04 |
| Maximum stent diameter, mm | 3.0 (2.8, 3.5) | 3.0 (3.0, 3.5) | 0.003 |
| Total stent length, mm | 33 (23, 52) | 29 (22, 44) | 0.07 |
| Post-dilation performed | 97 (91.5) | 327 (93.7) | 0.43 |
| Maximum balloon pressure, atm | 16 (16, 18) | 16 (16, 18) | 0.56 |
| Contrast media, mL | 150 (130, 180) | 150 (120, 180) | 0.06 |
| Procedural time, min | 35 (24, 59) | 30 (20, 47) | 0.02 |
| Procedural success# | 102 (96.2) | 345 (98.9) | 0.09 |
| Complete revascularization | 83 (78.3) | 320 (91.7) | <0.001 |
| Staged PCI for non-culprit lesions | 15 (14.2) | 20 (5.7) | 0.004 |
| **Baseline quantitative coronary angiography** | | | |
| Reference vessel diameter, mm | 2.64 (2.40, 2.93) | 3.05 (2.75, 3.45) | <0.001 |
| Minimal lumen diameter, mm | 0.75 (0.00, 1.12) | 1.10 (0.75, 1.48) | <0.001 |
| Diameter stenosis, % | 63.5 (54.1, 70.3) | 61.2 (52.1, 69.4) | 0.67 |
| Lesion length, mm | 26.2 (18.5, 41.8) | 25.8 (18.2, 38.8) | 0.48 |
| **Post-procedure quantitative coronary angiography** | | | |
| Reference vessel diameter, mm | 2.95 (2.70, 3.36) | 3.20 (2.81, 3.61) | 0.001 |
| Minimal lumen diameter, mm | 2.50 (2.21, 2.84) | 2.75 (2.37, 3.16) | <0.001 |
| Acute gain, mm | 1.75 (1.33, 2.39) | 1.70 (1.30, 2.21) | 0.20 |
| Diameter stenosis, % | 16.0 (10.7, 21.8) | 13.3 (7.3, 19.7) | 0.005 |

Binary data are expressed as n (%). Continuous data are expressed as mean ± standard deviation or median (interquartile range).

IVUS denotes intravascular ultrasound, PCI denotes percutaneous coronary intervention.

* The lesion most likely responsible for the acute coronary syndrome as determined by the operator.

† Defined as Medina 0,1,1 or 1,1,1 bifurcation lesion with a side branch ≥2·5 mm in diameter by visual estimation.

§ Defined as the lesion length of at least 30 mm in length by visual estimation.

¶ Defined as the angiographic presence of calcium on both sides of the vessel at the lesion site.

‖ Defined as an intraluminal filling defect seen in multiple projections.

# Defined as Thrombolysis In Myocardial Infarction (TIMI) flow grade 3, residual stenosis <20%, and absence of ≥type B dissection, with no intra-procedural complications.

**Table S5. Performance of the TVF-ACS Risk-Score among ACS patients in the IVUS-XPL trial**

|  | **High-risk**  **(n=303)** | **Low-risk**  **(n=383)** | **HR (95% CI)** | **P-value** |
| --- | --- | --- | --- | --- |
| TVF | 23 (7.6) | 11 (2.9) | 2.82 (1.37 – 5.78) | 0.005 |
| TVF without PMI | 23 (7.6) | 11 (2.9) | 2.82 (1.37 – 5.78) | 0.005 |
| All-cause death | 4 (1.3) | 1 (0.3) | 5.22 (0.58 – 46.68) | 0.14 |
| Cardiac death | 4 (1.3) | 1 (0.3) | 5.22 (0.58 – 46.68) | 0.14 |
| TV-MI | 0 (0.0) | 0 (0.0) | - | - |
| TVR | 20 (6.6) | 10 (2.6) | 2.57 (1.20 – 5.53) | 0.02 |
| TLR | 20 (6.6) | 10 (2.6) | 2.57 (1.20 – 5.53) | 0.02 |
| Stent thrombosis | 3 (1.0) | 0 (0.0) | - | - |

Data are number (%) of events (Kaplan-Meier estimated percentage) at 1-year.

IVUS = intravascular ultrasound; PMI = procedural myocardial infarction; TLR = target-lesion revascularization; TVF = target-vessel failure; TV-MI = target-vessel myocardial infarction; TVR = target-vessel revascularization.

**Table S6. Performance of the TVF-ACS Risk-Score among ACS patients in the ULTIMATE trial**

|  | **High-risk**  **(n=325)** | **Low-risk**  **(n=811)** | **HR (95% CI)** | **P value** |
| --- | --- | --- | --- | --- |
| TVF | 26 (8.0) | 24 (3.0) | 2.77 (1.59 – 4.82) | <0.001 |
| TVF without PMI | 20 (6.2) | 20 (2.5) | 2.52 (1.36 – 4.70) | 0.003 |
| All-cause death | 13 (4.0) | 10 (1.2) | 3.28 (1.44 – 7.48) | 0.005 |
| Cardiac death | 10 (3.1) | 5 (0.6) | 5.04 (1.72 – 14.75) | 0.003 |
| TV-MI | 8 (2.5) | 8 (1.0) | 2.51 (0.94 – 6.70) | 0.07 |
| - Procedural | 8 (2.5) | 4 (0.5) | 5.01 (1.51 – 16.63) | 0.009 |
| - Non-procedural | 0 (0.0) | 4 (0.5) | - | - |
| TVR | 10 (3.1) | 14 (1.7) | 1.81 (0.81 – 4.08) | 0.15 |
| TLR | 10 (3.1) | 11 (1.4) | 2.32 (0.98 – 5.46) | 0.054 |
| Stent thrombosis | 2 (0.6) | 4 (0.5) | 1.25 (0.23 – 6.81) | 0.80 |

Data are number (%) of events (Kaplan-Meier estimated percentage) at 1-year.

IVUS = intravascular ultrasound; PMI = procedural myocardial infarction; TLR = target-lesion revascularization; TVF = target-vessel failure; TV-MI = target-vessel myocardial infarction; TVR = target-vessel revascularization.

**Table S7. Model discrimination and calibration performance of various risk scores for one-year target vessel failure compared with the internal validation dataset from IVUS-ACS trial**

|  | **AUC (95% CI)** | **P value** | **NRI (95% CI)** | **P value** |
| --- | --- | --- | --- | --- |
| TVF-ACS Risk-Score | 0.709 (0.630, 0.788) | Reference | Reference | Reference |
| GRACE Score (original) | 0.567 (0.469, 0.665) | 0.004 | 0.21 (0.06, 0.36) | 0.007 |
| GRACE 2.0 score | 0.549 (0.450, 0.648) | 0.003 | 0.25 (0.05, 0.46) | 0.01 |
| SYNTAX score | 0.617 (0.532, 0.701) | 0.07 | 0.29 (0.08, 0.50) | 0.006 |
| DAPT score | 0.526 (0.445, 0.607) | <0.001 | 0.25 (0.03, 0.47) | 0.03 |
| PRECISE-DAPT score | 0.513 (0.410, 0.616) | 0.001 | 0.32 (0.11, 0.53) | 0.003 |

GRACE denotes Global Registry of Acute Coronary Events; DAPT, Dual Antiplatelet Therapy; PRECISE-DAPT, Predicting Bleeding Complications in Patients Undergoing Stent Implantation and Subsequent Dual Antiplatelet Therapy); SYNTAX, Synergy Between PCI With Taxus and Cardiac Surgery; NRI, net reclassification index.

**Table S8. β-values and assigned points for each variable in the TVF-ACS Risk-Score**

| **Variable** | **β-values** | **Points** |
| --- | --- | --- |
| Race | -1.681761637 | 6.146636957 |
| ST-segment elevation myocardial infarction | 0.460545621 | 1.683238975 |
| Peripheral arterial disease | 0.513571271 | 1.877041361 |
| History of spontaneous bleeding | 0.718390181 | 2.625629898 |
| Left ventricular ejection fraction | -0.234 | 0.86 |
| Low-density lipoprotein cholesterol | 0.0684 | 0.25 |
| Multivessel disease | 0.347411606 | 1.269747729 |
| Moderate or severe calcification | 0.499708319 | 1.826373936 |
| Thrombus-containing lesions | 0.694946599 | 2.539946418 |
| Reference vessel diameter | -0.059 | 0.216 |

**Table S9. the proportion of missing data for all candidate variables**

| Variable | Missing proportion | Variable | Missing proportion | Variable | Missing proportion |
| --- | --- | --- | --- | --- | --- |
| Age | 0 | Thrombus-containing | 0 | COPD | 0 |
| Sex | 0 | Transradial access | 0 | Anemia | 0 |
| Race | 0 | Aspiration thrombectomy used | 0 | Liver disease | 0 |
| Initial presentation | 0 | Rotational atherectomy used | 0 | Malignancy | 0 |
| Hypertension | 0 | Drug-eluting stent type | 0 | Left ventricular ejection fraction | 11.6% |
| Diabetes mellitus | 0 | Number of stents used | 0 | White blood cells | 0 |
| Insulin treatment | 0 | stent diameter | 0 | Red blood cells | 0 |
| Dyslipidemia | 0 | stent length | 0 | Hemoglobin | 0 |
| Current smoking* | 0 | Post-dilation performed | 0 | Platelet | 2.2% |
| Chronic kidney disease | 0 | balloon pressure | 0 | SGPT | 0 |
| Previous PCI | 0 | Contrast media | 4.3% | SGOT | 0 |
| Previous CABG | 0 | Procedural time | 3.9% | Serum creatinine | 1.8% |
| Previous myocardial infarction | 0 | Procedural success‖ | 0 | eGFR | 1.8% |
| Previous stroke | 0 | Complete revascularization | 0 | Fasting glucose | 0 |
| Peripheral arterial disease | 0 | Staged PCI for non-culprit lesions | 0 | Total cholesterol | 3.5% |
| Heart failure | 0 | Height | 0 | LDL-cholesterol | 3.8% |
| Number of diseased vessels | 0 | Weight | 0 | Aspirin | 0 |
| Total number of lesions treated | 0 | Systolic blood pressure | 0 | Ticagrelor | 0 |
| Culprit lesion location | 0 | Diastolic blood pressure | 0 | β blocker | 0 |
| True bifurcation | 0 | Heart rate, | 0 | ACEI or ARB | 0 |
| Long or diffuse | 0 | Family history of CAD | 0 | Calcium channel antagonist | 0 |
| Moderate or severe calcification | 0 | Previous spontaneous bleeding | 0 | Statin | 0 |

**Figure S1. Predictor selection using LASSO regression analysis with tenfold cross-validation.**

A) LASSO coefficient profile plot; B) Risk factors selected using LASSO regression; C) Forest plot of significant parameters in multivariable Cox regression analysis. LASSO, least absolute shrinkage and selection operator.


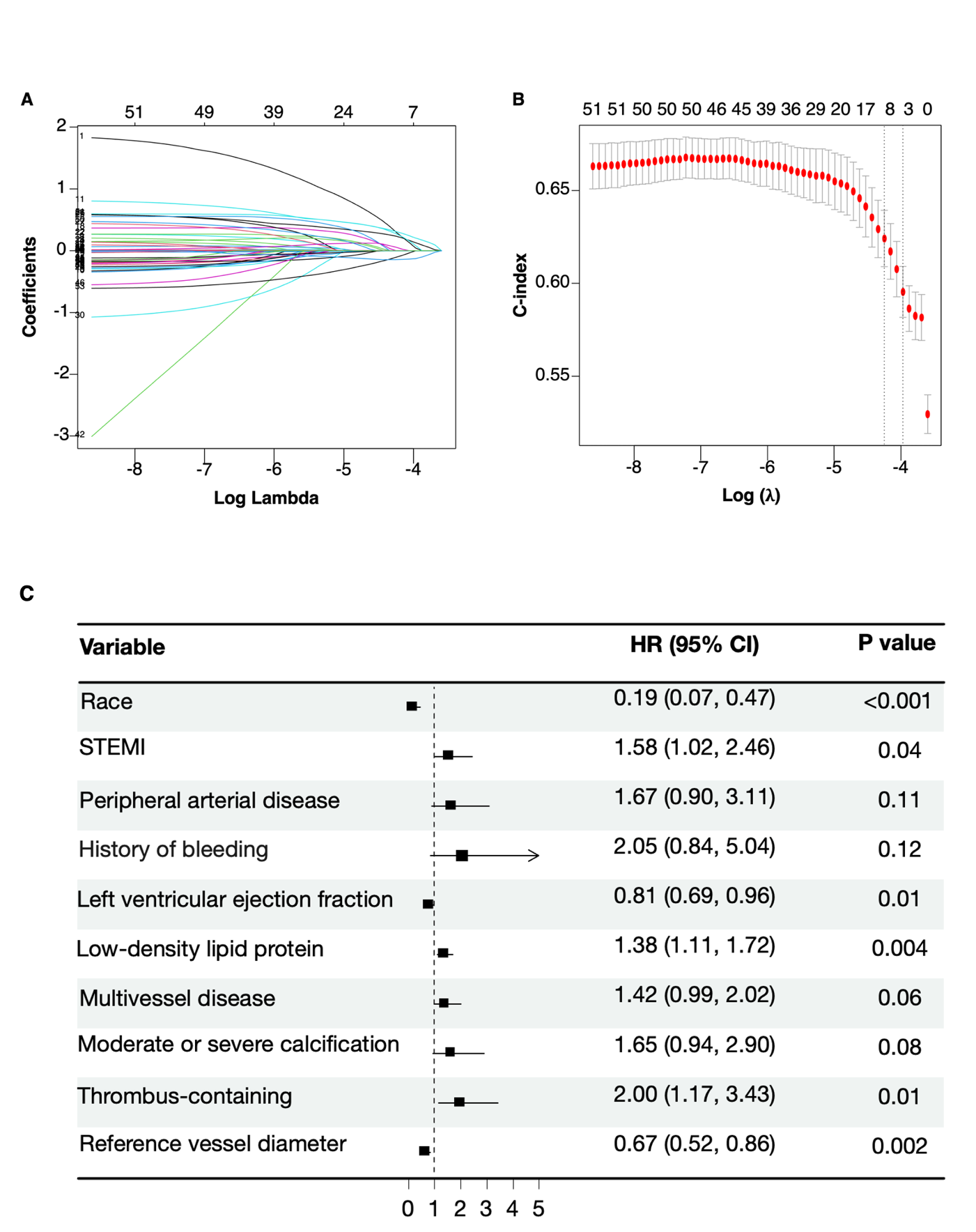


**Figure S2. Discrimination of the TVF-ACS Risk-Score for 1-year TVF.**

A) Derivation dataset; B) Validation dataset. AUC = area under the curve.


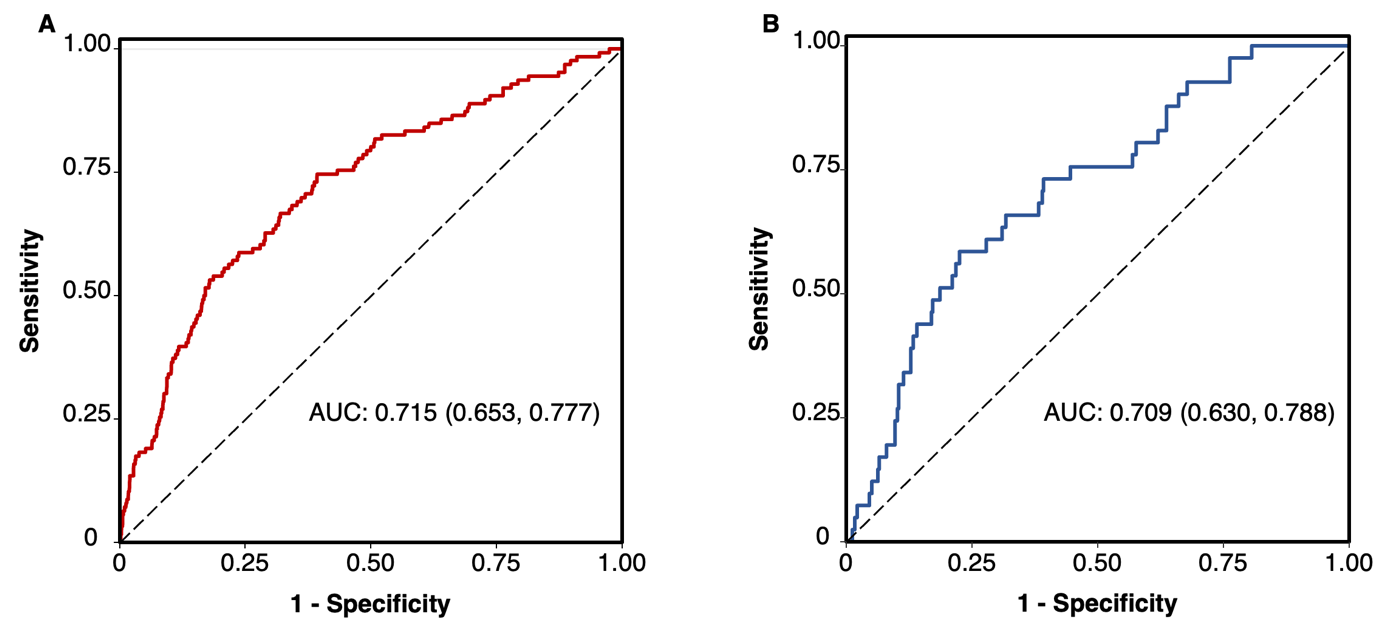


**Figure S3.** **Calibration of the TVF-ACS Risk-Score for 1-year TVF.**

A) Derivation dataset; B) Validation dataset. AUC = area under the curve.

**
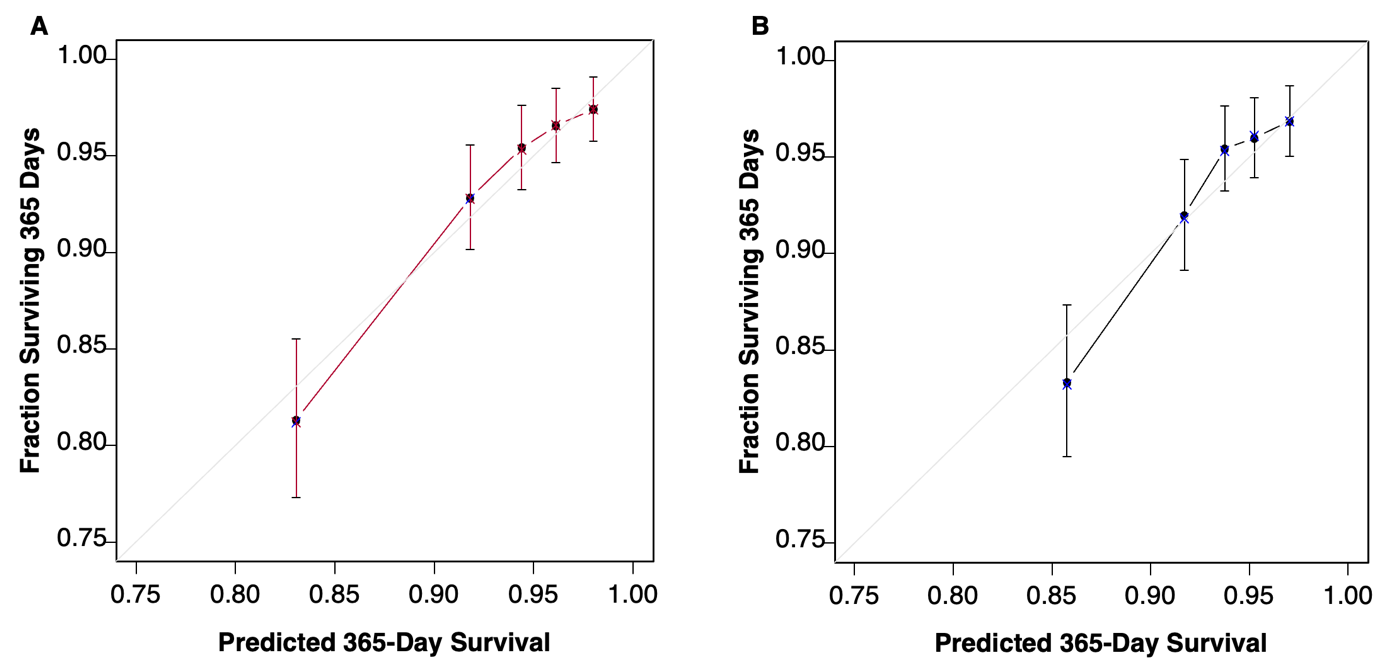
**

**Figure S4.** **Performance of the TVF-ACS Risk-Score in the IVUS-XPL trial.**

**
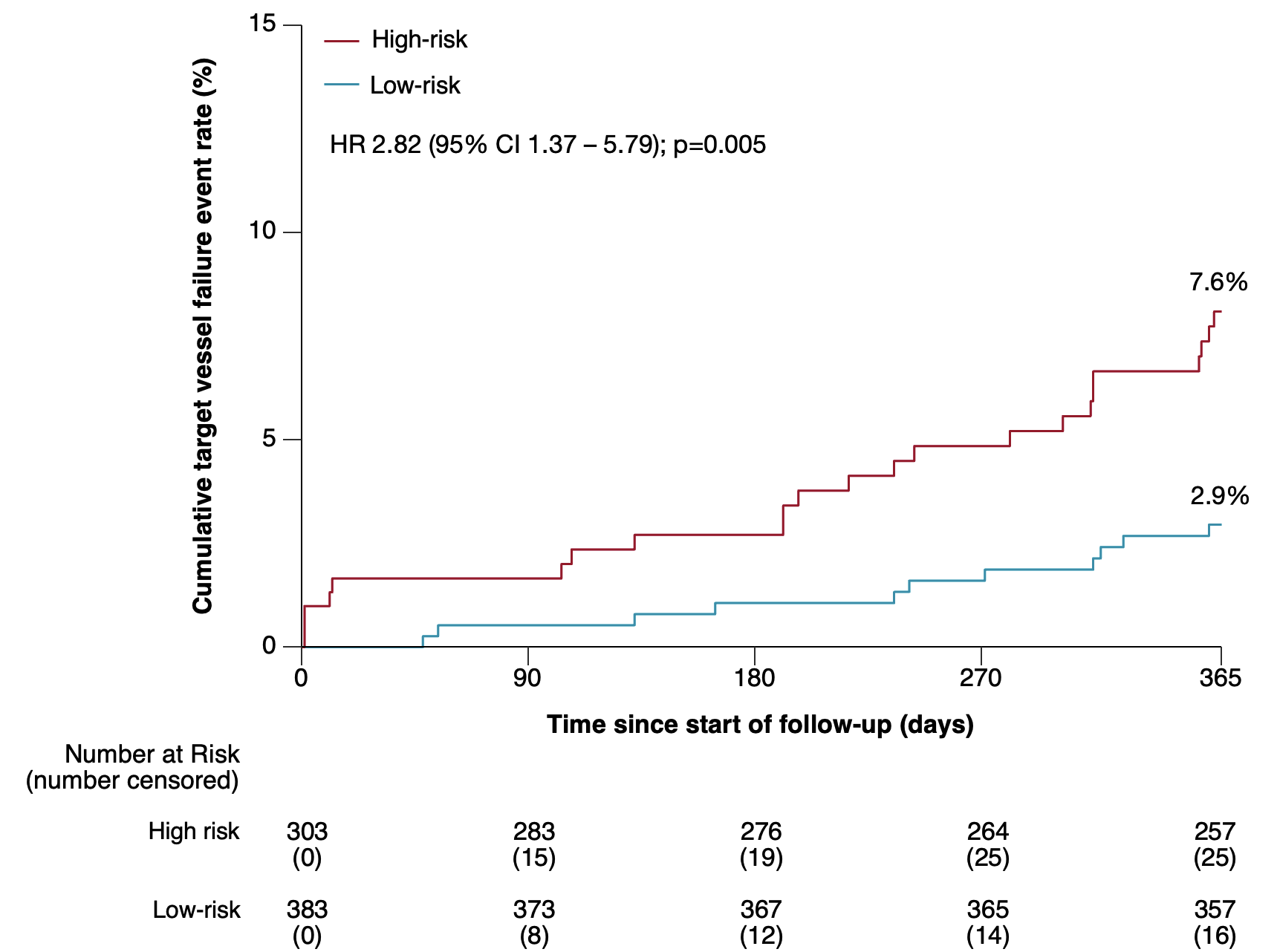
**

**Figure S5.** **Performance of the TVF-ACS Risk-Score in the ULTIMATE trial.**


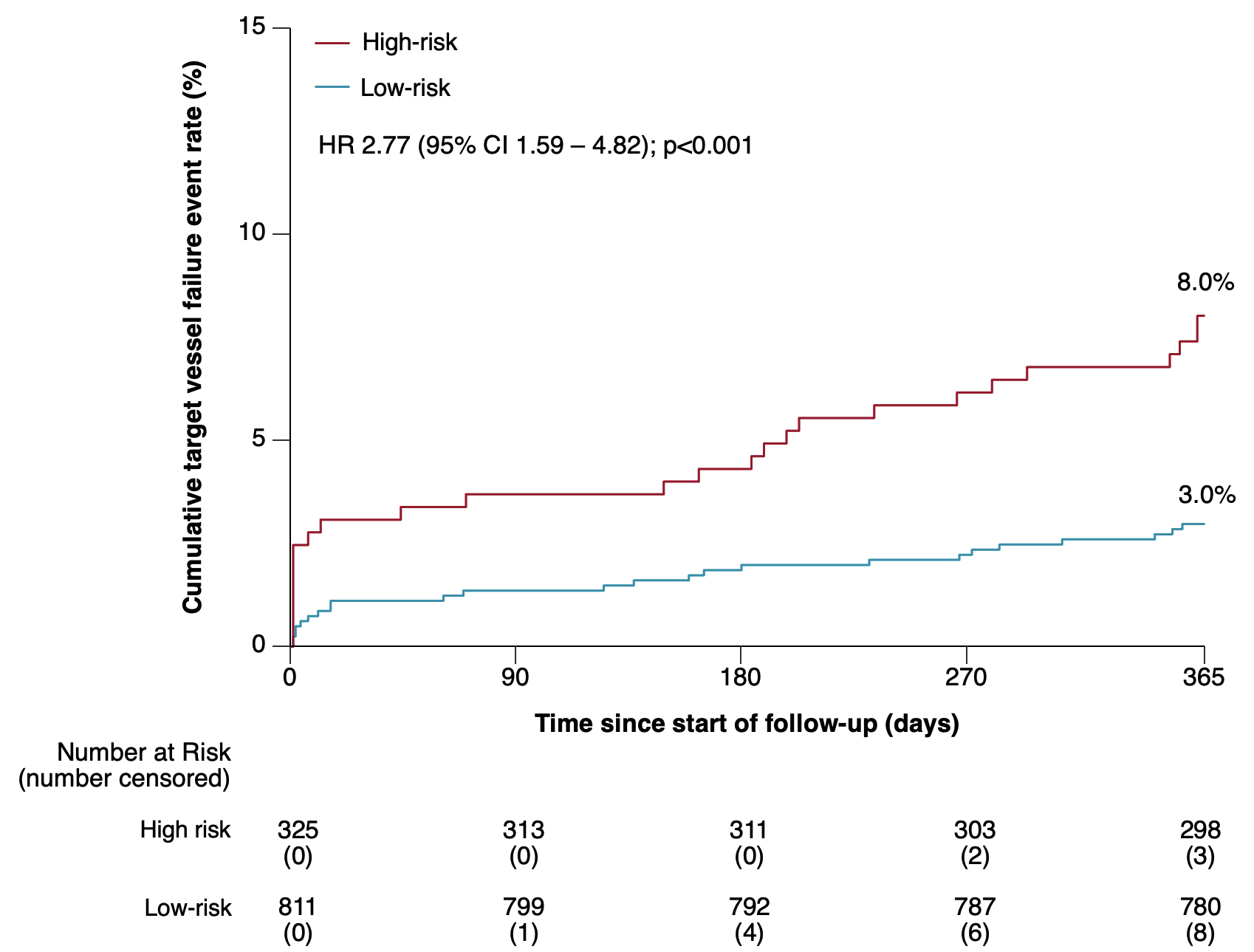


**Figure S6.** **One-year TVF in high-risk and low-risk patient groups randomized to angiography guidance vs. IVUS guidance in the IVUS-XPL trial.**

**
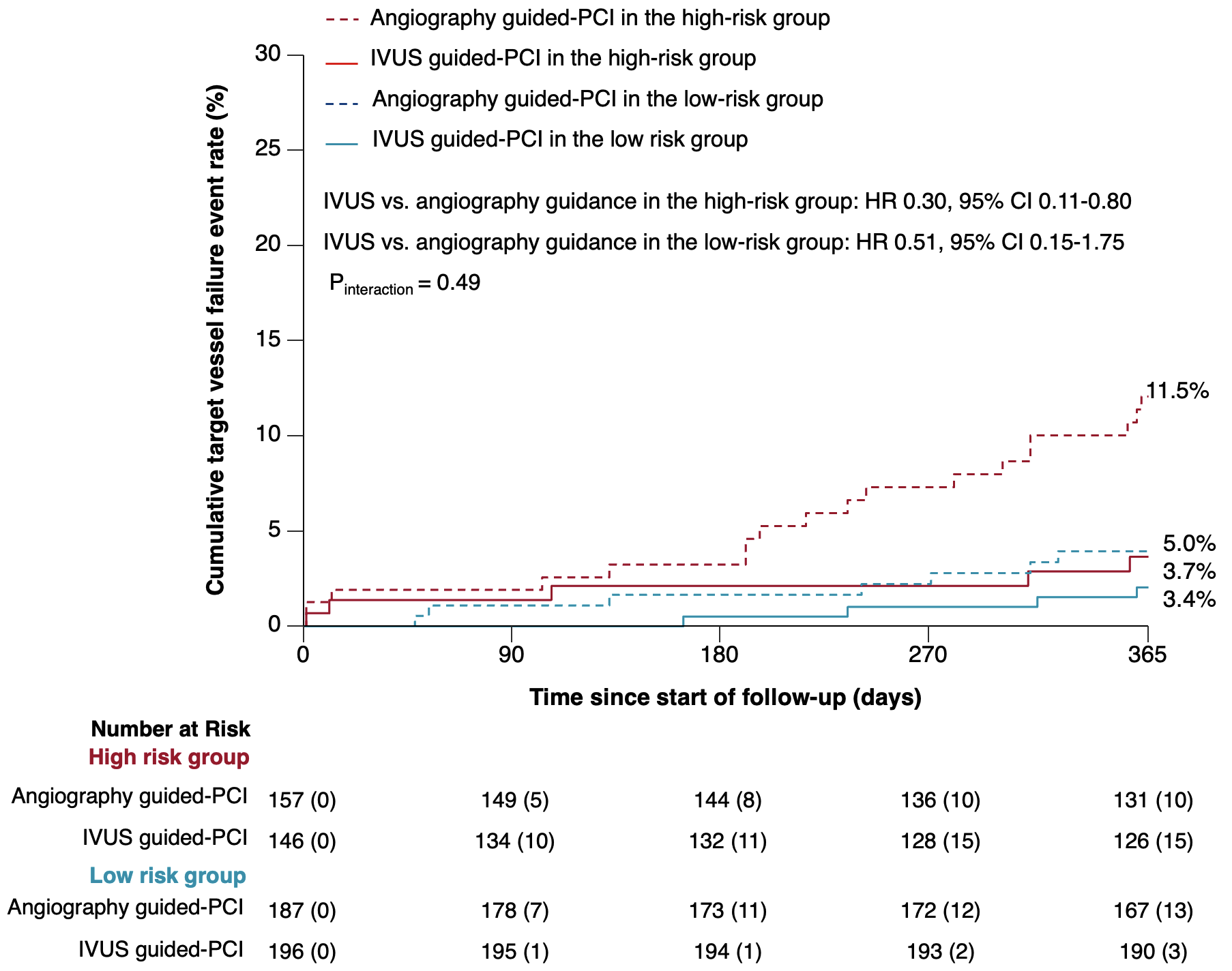
**

**Figure S7.** **One-year TVF in high-risk and low-risk patient groups randomized to angiography guidance vs. IVUS guidance in the ULTIMATE trial.**

**
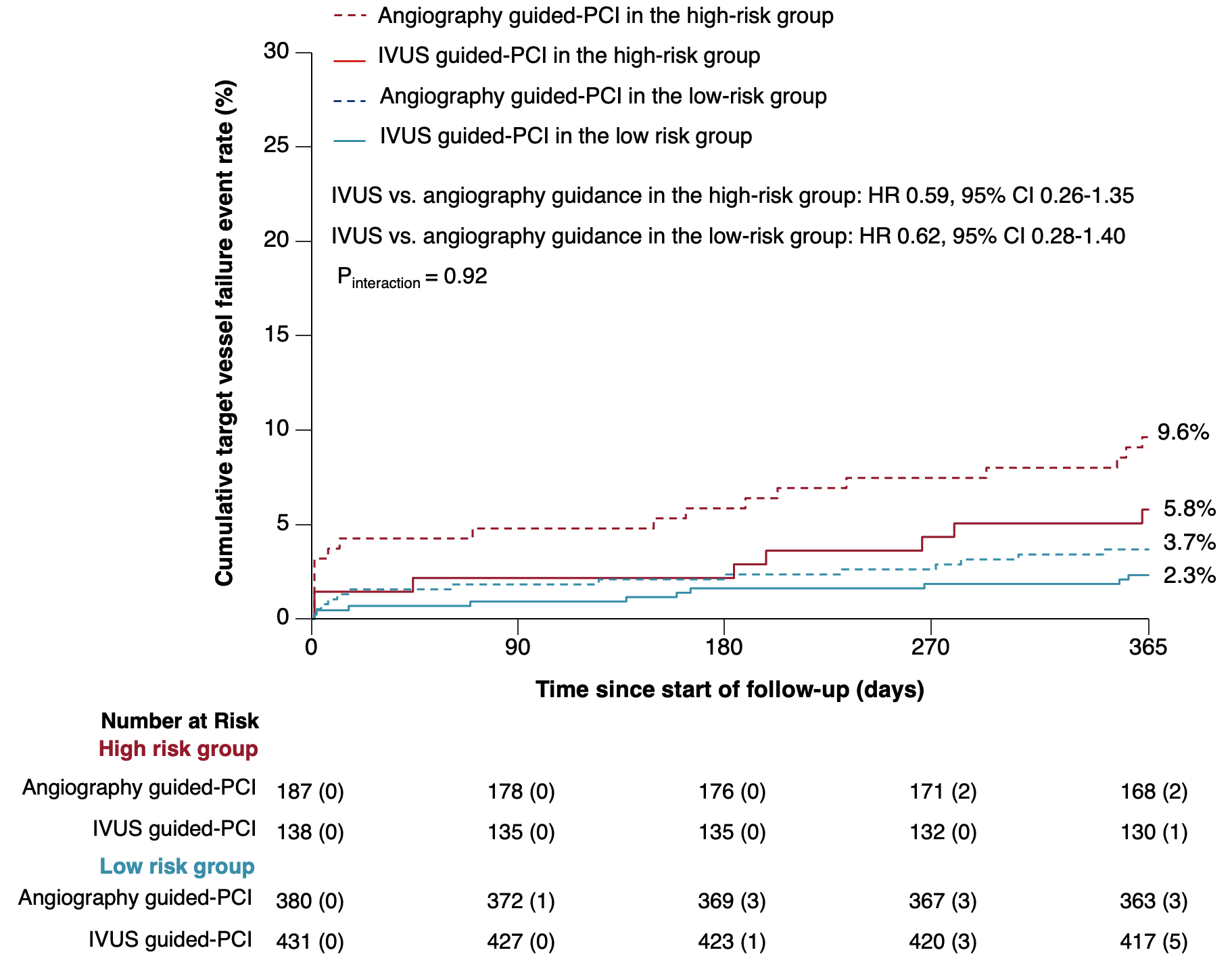
**
